# Study protocol: a multi-center, double-blind, randomized, 6-month, placebo-controlled trial to investigate the effect of supplementing hormone therapy FET cycles with Gushen’antai pills on the outcomes of in vitro fertilization

**DOI:** 10.1186/s13063-021-05614-w

**Published:** 2021-09-26

**Authors:** Ying-jie Ma, Xian-ling Cao, Ting Ma, Jing-yan Song, Ling-yu Yu, Yang-yang Yu, Jian-Yun Zhao, Zhen-Gao Sun

**Affiliations:** 1 Shandong University of Traditional Chinese Medicine, Jinan, 250011 China; 2 Integrative Medicine Research Centre of Reproduction and Heredity, The Affiliated Hospital of Shandong University of Traditional Chinese Medicine, No 42 Wen Hua Xi Road, Jinan, 250011 China

**Keywords:** FET, Hormone therapy, TCM, Randomized controlled trial, Ongoing pregnancy rate

## Abstract

**Background:**

Infertility is a widespread global challenge. Currently, the most effective treatment strategy for infertility is in vitro fertilization (IVF), which is an assisted reproductive technique (ART). The use of IVF for assisted pregnancy dates back to the last 41 years when the first IVF baby was born. During IVF, many oocytes are obtained in an IVF cycle, and more than one embryo is formed. Subsequently, frozen-thawed embryo transfer (FET) is increasingly being used in IVF cycles for women in whom a fresh embryo transfer fails to result in a pregnancy, or in those who return for a second baby. However, the pregnancy success rates following FET treatment cycles are reportedly lower than in fresh embryo transfers. Therefore, recent related studies are increasing determining mechanisms of improving the sustained pregnancy rate of FET and reducing the rate of early abortion.

The Gushen’antai pill (GSATP), which contains a mixture of 10 herbs, has been widely used in traditional Chinese medicine (TCM) as a pharmacological option to prevent miscarriage. However, randomized controlled trials (RCT) have never been conducted to provide high-level clinical evidence on the clinical efficacy of GSATP. The objective of this study is to investigate the effect of GSATP of hormone therapy (HT) FET cycles on pregnancy rate.

**Methods:**

A total of 300 subjects aged between 18 and 40 years which prepared for HT cycle FET will be enrolled in the study. The patients were from five different hospitals, with 60 patients from each hospital. Patients were randomly divided into two groups, and medication was started on the day of endometrial transformation. After FET 28 days, B-ultrasound was done to determine whether to continue the medication. Baseline assessments were carried out before the trial and outcomes were collected 4, 6, 8, 10, and 12 weeks of each gestational cycle.

**Discussion:**

Differences in ongoing pregnancy rate, clinical pregnancy rate, implantation rate, and threatened abortion rate between the two groups will be statistically analyzed. We can finally have an objective evaluation of the efficacy of the traditional Chinese medicine Gushen’antai pills.

**Trial registration:**

ChiCTR1900026737. Registered October 20, 2019.

## Background

Infertility is a global challenge estimated to affect between 8 and 12% of couples in childbearing age worldwide [1]. Being an effective treatment, IVF, an ART has been widely used for the last 41 years since the first IVF baby was born in 1978. Previously, IVF was mainly done by the transfer of fresh embryos. In recent years, however, FET has increasingly gained importance in IVF protocols [2].

With the increasing number of FET cycles, an assessment of the best endometrial preparation is primary to maximizing the success rate of ART [3]. Also, FET helps to maximize the cumulative pregnancy rate per oocyte retrieval. Previous clinical studies have reported that the pregnancy rate in FET cycles is lower than fresh IVF/ intracytoplasmic sperm injection (ICSI) cycles [4]. However, the latest large retrospective cohort study found no significant difference between FET cycles and fresh cycles [5]. Besides, the increasing concerns on the adverse effects of controlled ovarian stimulation (COS) on the endometrial and uterine environment have increased the popularity of FET [6]. Current studies have mostly focused on different cycle regimens, such as natural cycles and HT cycles, for the endometrial preparation of FET [7]. While the natural ovulatory cycles are spontaneous, HT cycles use estrogen and progesterone hormones to prepare endometrium and are, therefore, more precise.

Although the results of a meta-analysis revealed that the type of estrogen supplementation and the route of administration do not affect the success rate of FETs [8], the effect of administration route and dose of progesterone is still controversial [9, 10]. Interestingly, no efficiency and safety differences between natural cycle FET and HT cycle FET could be found by a meta-analysis [11], which was also proven in another study [7, 12]. In a study performed by Givens et al., higher miscarriage rates were found in the HT cycle FET group, and there was also no difference between the live birth rates [13].

Gushen’antai pills (GSATP) are processed by TCM and are a classic prescription with modern pharmaceutical technology. The pill is a mixture of 10 herbs and has been widely used as a pharmacological option in China for preventing miscarriage [14, 15]. Notably, GSATP is approved by the Chinese State Food and Drug Administration (SFDA). According to the theory of TCM, a full kidney Qi is representative of strong reproductive ability, while the kidney Qi is exuberant and thus to keep the mother and fetus in a stable state. The GSATP is mainly used for tonifying the kidney, and the herbal components are mutually reinforcing. The pill is made up of ten kinds of precious Chinese herbal medicines (*Polygonum multiflori*, *Rehmannia glutinosa*, *Cistanche deserticola*, *Tripterygium wilfordii*, *Tripterygium wilfordii*, *Radix Atractylodes*, *Radix macrocephala*, *Radix Scutellariae*, *Radix Paeoniae*, *Rubra*, *Chinese dodder seed*, *Uncaria*, and *Mulberry Parasitoids*) through long-term clinical practice and optimized compatibility. According to the TCM theory, GSATP prevents miscarriage. The safety of TCM can be improved through strict syndrome differentiation and deep processing [16], and it is what GSATP pursues and do. No adverse drug reaction (ADR) has been reported during the many years of GSATP use.

Improving the success rate of the HT cycle FET is a complex process that has not yet been fully investigated. Therefore, multi-target therapy, such as TCM, may offer unique advantages in this complex disease treatment over the single-target use prevalent in western medicine [17].

Although the response of GSATP in patients is excellent, the lack of high-quality, evidence-based medicine restricts its widespread use. The combination of evidence-based medicine, modern medicine, and TCM is an exciting field that has continuously attracted immense attention and efforts [18]. Therefore, a large multi-center, double-blind, randomized, placebo-controlled trial should be conducted to develop a simple, inexpensive, and widely practical oral medication treatment based on GSATP.

### Aims

#### The primary objective and secondary objectives

The primary aim of this trial is to evaluate and verify the clinical efficacy of GSATP, and its effect on the ongoing pregnancy rate (OPR) of HT FET cycles. The secondary objectives are to determine whether GSATP affects implantation rate (IR), clinical pregnancy rate (CPR), and threatened abortion rate (TAR) of HT cycle FETs.

## Methods

### Diagnostic criteria

The GSATP is suitable for early threatened abortion (TA), which belongs to kidney yin deficiency in TCM. Early TA refers to the premonitory abortion within 12 weeks of pregnancy, which is mainly characterized by a small amount of vaginal bleeding, paroxysmal lower abdominal pain or low back pain, unopened cervical opening, undamaged fetal membrane, and the size of the uterus is consistent with the gestational week [19]. Kidney yin deficiency syndrome in TCM can be diagnosed as long as the waist and knees are sour and soft and any other one of the following symptoms: falling pain in the lower abdomen, or accompanied by dizziness and tinnitus, dry mouth and dry throat, mental fatigue, and feverish palms and soles.

### Recruitment

**Fig. 1:**
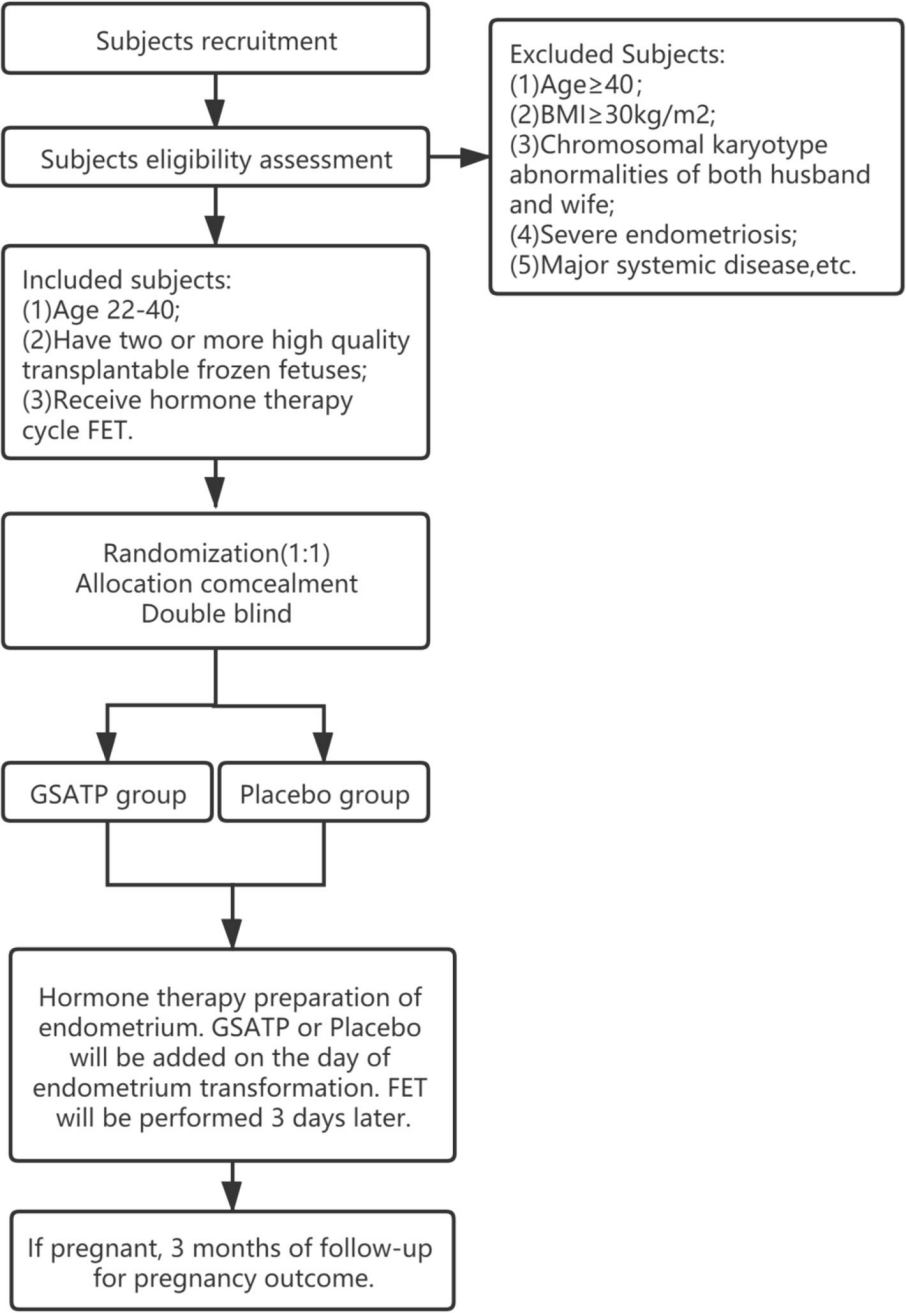
Study flow chart

We recruited participants who purposed to do FET from reproductive centers in five different tertiary hospitals: Affiliated Hospital of Shandong University of Traditional Chinese Medicine, Maternity and Child Health Care of ZaoZhuang, Shanxi Maternal and Child Health Care Hospital, The First Affiliated Hospital of Wenzhou Medical University, and Jinan Military General Hospital. These five hospitals are all tertiary A public hospitals and have the qualification of IVF-FET, recruiting 60 patients in each hospital. In order to ensure the consistency of the procedures in all the sites, the training was uniformly conducted before the start of the trial. During the trial, all the sites communicated in a timely manner through the network, and the progress was regularly checked by professionals to ensure the consistency and uniformity of the trial. This study was conducted according to the Declaration of Helsinki and approved by the Reproductive Medicine Ethics Committee of the Affiliated Hospital of Shandong University of Traditional Chinese Medicine (approval number 20191109). Written informed consent was obtained from patients before study enrollment. Prospective baseline data of patients were collected, including height, weight, basic endocrine level, and detailed IVF process record. Informed consent was obtained from the patients before any study procedures were done. We estimate that it will take about six months to complete this phase of the test. A study flow chart is displayed in Fig. 1.

### Inclusion criteria

Patients who met the following inclusion criteria were included in the study:

① Age: 22–40 years old
② Patients who had two or more high-quality transplantable frozen fetuses
③ Patients who received HT cycle FET
④ Patients who had less than three previous FET cycles, ≤two unexplained abortions, ≤ two implant failures
⑤ Patients who have not received similar drug treatment
⑥ Patients who have no history of mental illness and have no abnormality in liver and kidney function and electrocardiogram

### Exclusion criteria

The following was the exclusion criteria used in this study:

① BMI ≥ 30 kg/m^2^
② Patients with abnormal development of the reproductive system and one abnormal chromosome karyotype of both male and female
③ Patients with major systemic diseases
④ Patients with endometriosis, adenomyosis, and hydrosalpinx
⑤ Patients with previous endometriosis

### Allocation concealment mechanism

Participants were randomly assigned to either the control group or the experimental group, at a proportion of 1:1 according to the computer-generated random schedule in R software by an assessor who was blinded to the treatment allocation. Assignment concealment was guaranteed because our experiment identified the subjects from the start of the test. The uncertainty of subjects can fully guarantee the effect of allocation concealment.

Clinical recovery was evaluated by an assessor who was blinded to the treatment allocation. The assessor was comprehensively trained before the study was done. Because of the professionalism of the medical profession, neither the participants nor the staff was assigned blindly and naturally, but the distribution of the participants was not disclosed in subsequent evaluations. Patient-related information such as OPR, CPR, IR, and TAE was collected by personnel who were not directly involved in the experiment. The doctors only collected the information at the end of the trial.

### Potential risks and handling

Although GSATP has a safe history of pharmacological use, we do not rule out that its use could cause some side effects. At present, the commonly reported adverse reactions of TCM use mainly include gastrointestinal reactions, liver, and kidney damage. Subsequently, patients were well informed of the potential risks in advance.

### Patient and public involvement

Patients are neither involved in the development of research issues nor in the design and recruitment of research. However, patients participated in the study and were randomly divided into two groups, taking GSATP and placebo respectively. The cost of the drugs used in this study is borne by the research group, and the patients have no part of their own expenses.

The results of the study will be disseminated to participants and their families through WeChat notifications, patient organization platforms, and public information meetings

### Interventions

A randomized, double-blind control method was used to select eligible patients according to the ratio of 1:1 between GSATP and placebo. The GSATP (Product specification: 6 g* 9 bags) and placebo (starch as an ingredient, without the active ingredients) were obtained from Beijing bran Pharmaceutical Inc and re-labeled by Beijing bran Pharmaceutical Inc, according to the national drug standards of the State Food and Drug Administration of China. In this paper, the application research of GSATP in the treatment of threatened abortion is consistent with the indications in its published instructions.

The routine dose of GSATP (6 g tid) or placebo (6 g tid) was orally administered to patients four hours before the endometrial transformation. We ask patients to take their medicine on time every day. Clinical pregnancy was confirmed by interpreting B-ultrasound results 28 days after FET. Oral administration of the treatment on pregnant women was continued until 12 weeks of pregnancy, but drug administration was not done on patients who had not conceived. Patients with ectopic pregnancy were included in adverse events. Both doctors and patients were blinded to the identity of the syringe contents. Patients were allowed to use other drugs in case of emergency, but data from such patients were not included in the statistical analysis.

Adherence reminder sessions were done during the initial product dispensing and at each follow-up visit. The following was communicated to the patients beforehand: the importance of adhering to the daily study product instructions, including the time taken, the importance of storing the study drug appropriately and making the entire study drug, and the measures to be taken in case of missed doses. One of our staff regularly contacted the patients via WeChat to enquire about the medication.

### Safety assessment

All subjects were managed and followed up by medical personnel including the chief physician during medication. Follow-up was performed in the outpatient department at weeks 2, 5, 7, 9, and 10 after FET to record any local and/or systemic reactions during medication and any adverse events throughout the study. Due to the wide range of targets for TCM, potential beneficial or adverse reactions in patients will also be recorded during each follow-up visit. All local and systemic adverse events, regardless of their severity, will be recorded. Once any adverse reaction occurs during medication, the drug will be stopped immediately and medical personnel will observe and deal with it according to the condition. The patient's questionnaire was used to collect data on other medication-related symptoms.

### Outcome measures

The primary endpoint was OPR at week 12 of pregnancy. The secondary endpoints were IR, CPR, and TAR. The OPR, IR, CPR, and TAR were measured at the end of the experiment. Hormone levels were monitored and recorded at FET on days 1, 7, and 14. IR (%) = number of implantation gestational sac/number of transferred embryo × 100%, CPR(%) = number of clinical pregnancy/number of transfer cycle × 100%, OPR(%) = number of continuous pregnancy/number of transfer cycle × 100%, and TAR(%) = number of abortion/number of clinical pregnancy × 100% (Table 1).

**Table 1:** Checklist of items for reporting trials of Chinese herbal medicine formulas

Section/topic	Item number	Standard CONSORT checklist item	Extension for CHM formulas	Reported on page number
**Title, abstract, and keywords**	1a	Identification as a randomized trial in the title	Statement of whether the trial targets a TCM pattern, a Western medicine-defined disease, or a Western medicine-defined disease with a specific TCM pattern, if applicable	1–4;19–49;52
	1b	Structured summary of trial design, methods, results, and conclusions (for specific guidance, see CONSORT for abstracts [26, 27])	Illustration of the name and form of the formula used, and the TCM pattern applied, if applicable	39–45;86–94
	1c		Determination of appropriate keywords, including “Chinese herbal medicine formula” and “randomized controlled trial”	52
**Introduction**				
**Background and objectives**	2a	Scientific background and explanation of rationale	Statement with biomedical science approaches and/or TCM approaches	55–95
	2b	Specific objectives or hypotheses	Statement of whether the formula targets a Western medicine–defined disease, a TCM Pattern, or a Western medicine-defined disease with a specific TCM Pattern	96–99;106–111
**Methods**				
Trial design	3a	Description of trial design (such as parallel, factorial), including allocation ratio		156–161
	3b	Important changes to methods after trial commencement (such as eligibility criteria), with reasons		Not applicable
Participants	4a	Eligibility criteria for participants	Statement of whether participants with a specific TCM Pattern were recruited, in terms of (1) diagnostic criteria and (2) inclusion and exclusion criteria. All criteria used should be universally recognized, or reference given to where detailed explanation can be found.	113–122; 137–144
	4b	Settings and locations where the data were collected		124–128
Interventions	5	The interventions for each group with sufficient details to allow replication, including how and when they were actually administered	Description(s) for different types of formulas should include the following: 5a. **For fixed CHM formulas** 1. Name, source, and dosage form (e.g., decoctions, granules, powders) 2. Name, source, processing method, and dosage of each medical substance. Names of substances should be presented in at least 2 languages: Chinese (Pinyin), Latin, or English. Names of the parts of the substances used should be specified. 3. Authentication method of each ingredient and how, when, where, and by whom it was conducted; statement of whether any voucher specimen was retained, and if so, where they were kept and whether they are accessible 4. Principles, rationale, and interpretation of forming the formula 5. Reference(s) as to the efficacy of the formula, if any 6. Pharmacologic study results of the formula, if any 7. Production method of the formula, if any 8. Quality control of each ingredient and of the product of the formula, if any. This would include any quantitative and/or qualitative testing method(s); when, where, how, and by whom these tests were conducted; whether the original data and samples were kept, and, if so, whether they are accessible. 9. Safety assessment of the formula, including tests for heavy metals and toxic elements, pesticide residues, microbial limit, and acute/chronic toxicity, if any. If yes, it should be stated when, where, how, and by whom these tests were conducted; if the original data and samples were kept; and, if so, whether they are accessible. 10. Dosage of the formula, and how the dosage was determined 11. Administration route (e.g., oral, external) **5b. For individualized CHM formulas** 1. See recommendations 5a 1–11 2. Additional information: how, when, and by whom the formula was modified **5c. For patent proprietary CHM formulas** 156–163 1. Reference to publicly available materials, such as pharmacopeia, for the details about the composition, dosage, efficacy, safety, and quality control of the formula 2. Illustration of the details of the formula, namely (1) the proprietary product name (i.e., brand name), (2) name of manufacturer, (3) lot number, (4) production date and expiry date, (5) name and percentage of added materials, and (6) whether any additional quality control measures were conducted 3. Statement of whether the patent proprietary formula used in the trial is for a condition that is identical to the publicly available reference **5d. Control groups 156–161** Placebo control 1. Name and amount of each ingredient 2. Description of the similarity of placebo with the intervention (e.g., color, smell, taste, appearance, packaging) 3. Quality control and safety assessment, if any 4. Administration route, regimen, and dosage 5. Production information: where, when, how, and by whom the placebo was produced Active control 1. If a CHM formula was used, see recommendations 5a–5c 2. If a chemical drug was used, see item 5 of the CONSORT Statement (24)	86–92; 162–176; 201–202 Since GSATP is a national class III new drug approved according to the new drug registration standard, its specific content is confidential, so it cannot be published. The drug instructions can be queried at the address provided in the article. The rest are not applicable.
Outcomes	6a	Completely defined, prespecified primary and secondary outcome measures, including how and when they were assessed	Illustration of outcome measures with Pattern in detail	187–194
	6b	Any changes to trial outcomes after the trial commenced, with reasons		Not applicable
Sample size	7a	How sample size was determined		196–202
	7b	When applicable, explanation of any interim analyses and stopping guidelines		179–188
Randomization				
Sequence	8a	Method used to generate the random allocation sequence		220
	8b	Type of randomization; details of any restriction (such as blocking and block size)		220–224
Allocation concealment mechanism	9	Mechanism used to implement the random allocation sequence (such as sequentially numbered containers), describing any steps taken to conceal the sequence until interventions were assigned		225–232
Implementation	10	Who generated the random allocation sequence, who enrolled participants, and who assigned participants to interventions		222; 226–232
Blinding	11a	If done, who was blinded after assignment to interventions (for example, participants, care providers, those assessing outcomes) and how		230–233
	11b	If relevant, a description of the similarity of interventions		164–165
Statistical methods	12a	Statistical methods used to compare groups for primary and secondary outcomes		205–212
	12b	Methods for additional analyses, such as subgroup analyses and adjusted analyses		Not applicable
**Results**				
Participant flow (a diagram is strongly recommended)	13a	For each group, the numbers of participants who were randomly assigned, received intended treatment, and were analyzed for the primary outcome		This is only a research protocol, and the subjects have not been recruited, so this item is not applicable.
	13b	For each group, losses and exclusions after randomization, together with reasons		This is only a research protocol, and the subjects have not been recruited, so this item is not applicable.
Recruitment	14a	Dates defining the periods of recruitment and follow-up		135–136; 185–187
	14b	Why the trial ended or was stopped		167–169; 186–188
Baseline data	15	A table showing baseline demographic and clinical characteristics for each group		This is only a research protocol, and the subjects have not been recruited, so this item is not applicable.
Numbers analyzed	16	For each group, number of participants (denominator) included in each analysis and whether the analysis was by original assigned groups		This is only a research protocol, and the subjects have not been recruited, so this item is not applicable.
Outcomes and estimation	17a	For each primary and secondary outcome, results for each group, and the estimated effect size and its precision (such as 95% confidence interval)		This is only a research protocol, and the subjects have not been recruited, so this item is not applicable.
	17b	For binary outcomes, presentation of both absolute and relative effect sizes is recommended		Not applicable.
Ancillary analyses	18	Results of any other analyses performed, including subgroup analyses and adjusted analyses, distinguishing prespecified from exploratory		Not applicable.
Harms	19	All important harms or unintended effects in each group (for specific guidance, see CONSORT for harms [28])	(There is no extension for this item)	Not applicable.
**Discussion**				
Limitations	20	Trial limitations; addressing sources of potential bias; imprecision; and, if relevant, multiplicity of analyses		271–274
Generalizability	21	Generalizability (external validity, applicability) of the trial findings	Discussion of how the formula works on different TCM Patterns or diseases	Not Applicable.
Interpretation	22	Interpretation consistent with results, balancing benefits and harms, and considering other relevant evidence	Interpretation with TCM theory	Not applicable.
**Other information**				
Registration	23	Registration number and name of trial registry		50
Protocol	24	Where the full trial protocol can be accessed, if available		Not applicable.
Funding	25	Sources of funding and other support (such as supply of drugs), role of funders		292–294

*CHM* Chinese herbal medicine, *CONSORT* Consolidated Standards of Reporting Trials, *TCM* traditional Chinese medicine

The original CONSORT items are provided; elaborations for CHM formulas are in italicized text. We strongly recommend reading this checklist in conjunction with the CONSORT 2010 Explanation and Elaboration [29] for important clarifications on all original items of CONSORT Statement

### Statistical analysis

#### Sample size

The sample size was calculated based on a primary study [20], that revealed the rate of ongoing pregnancy in the observation group was significantly higher than that of the control group (63.83% VS 42.86%, *x*^2^=4.24, *P*< 0.05). An estimated OPR of 0.6 was obtained. According to 1:1 ratio, 65% efficacy of GSATP, *α*=0.05 and *β*=0.2, there were 130 patients in the treatment group and 130 in the placebo group. Considering the complexity of clinical practice, the loss rate is estimated to be 0.1. Finally, the sample size was calculated to be 300 subjects.

#### Analysis

The study data were collected and managed by non-clinical staff who were responsible for data management in each clinical center. The data were shown as follows: continuous variables with normal distribution were presented as means±SD, and the count data are presented in the form of n (%). Statistics were run using SPSS version 21 software (SPSS, Inc., Chicago, IL). The differences between the two groups were detected using *χ*^2^ for counting data or t test used for comparative analysis of measuring data. *P* values of less than 0.05 were considered to be statistically significant.

## Discussion

To our knowledge, this is the first study protocol to investigate the efficacy and safety of GSATP as an adjuvant drug for the outcomes of IVF, compared to a placebo treatment. Although FET increases the cumulative pregnancy rate, reduces costs, and is relatively simple and time-saving compared with repeated IVF or ICSI cycles with fresh embryo transfer, the pregnancy rate after the treatment cycle of FET has been considered to be lower than that of fresh embryo transfer [11]. The advantages of HT are high control and flexibility of transfer time and the length of follicular phase can be changed without affecting the IR or CPR. However, studies have found that the HT FET cycle may be related to adverse maternal and neonatal outcomes, such as hypertensive disorders of pregnancy and high risk of low birth weight infants [21, 22]. Although GSATP adjuvant therapy for HT FET cycles has been proved to be beneficial, the evidence of therapeutic effect is limited to small sample size or short-term follow-up [20]. Hence, a larger sample size randomized double-blind placebo-controlled trial is needed to verify its exact effect.

According to the theory of TCM, “Kidney Dominating Reproduction,” female infertility is closely related to kidney deficiency, and its main treatment principle is tonifying the kidney. According to the syndrome differentiation of TCM, A retrospective study of 485 patients with threatened abortion found that the distribution of TCM syndrome types is mainly kidney deficiency, which is also consistent with the theory of TCM [23]. GSATP is a change from the classic prescription of TCM. Its main function of traditional Chinese medicine is to tonify the kidney and prevent pregnancy loss. GSATP has been clinically applied for more than ten years since the approval of China’s State Food and Drug Administration and has achieved a good curative effect.

Modern pharmacological studies have found that Chinese medicine Uncaria and Mulberry Parasitoids have an antihypertensive effect, so they have a good effect on hypertensive disorders of pregnancy [24, 25], which is one of the side effects of HT FETcycle mentioned above. In addition, Chinese dodder seed can promote the secretion of hCG from placenta and promote embryonic development by significantly improve ovarian endocrine function [26]. It is clinically found that some people will have symptoms of constipation during pregnancy, and some components of GSATP can alleviate constipation [27]. Therefore, we will also follow up the remission of constipation during the experiment.

This study is a multi-center double-blind clinical trial with some limitations. The trial will not completely rule out clinical treatment bias. It will be difficult to guarantee that patients have similar backgrounds when comparing the two groups. In addition, the 12-week follow-up period completed in this study was limited and longer follow-ups are needed later to provide more reliable results.

In conclusion, the results of this study are expected to provide high-quality methodological evidence for the effectiveness and safety of GSATP adjuvant treatment of HT FET cycles.

## Ethics and dissemination

Financial support for the current study was provided by the National Natural Science Foundation of China (NSFC: 81874484) and approved by the reproductive medicine ethics committee of the Affiliated Hospital of Shandong University of TCM.

Written informed consent was obtained from the patients before the study was performed. Data was processed, recorded, and stored following the guidelines given by our ethics committee. Our research results will be published in various peer-reviewed journals and presented at scientific conferences.

### Strengths and limitations of this study

1. This trial was aimed at determining the clinical benefits and statistical significance of GSATP supplementation in the HT cycle FET’s treatment of preventing miscarriage.
2. This trial is not only the first multi-center double-blind RCT on GSATP but also a scientific evaluation of the actual clinical efficacy of GSATP.
3. This trial could provide an excellent demonstration and incentive for the modern scientific research of Chinese patent medicine on the significance of not violating the theory of TCM.
4. The 12-week follow-up period done in this study is limited, and longer follow-up could provide more reliable conclusions.
5. Multi-center studies are time-consuming, physically tasking, costly, and may, therefore, not be suitable for many TCM researchers.

## Data dissemination

The findings of this study will be widely disseminated through conference papers, research reports, and academic publications.

## Data Availability

All data generated during and/or analyzed during the study will be presented within the manuscript, and other detailed data are available from the corresponding author on reasonable request.

## Abbreviations

FET: Frozen-thawed embryo transfer
IVF: In vitro fertilization
ART: Assisted reproductive technique
GSATP: Gushen’antai pill
HT: Hormone therapy
COS: Controlled ovarian stimulation
ICSI: Intracytoplasmic sperm injection
RCT: Randomized controlled trials
CSFDA: Chinese state food and drug administration
ADR: Adverse drug reaction
TCM: Traditional Chinese medicine
